# Revealing the Appetite of the Marine Aquarium Fish Trade: The Volume and Biodiversity of Fish Imported into the United States

**DOI:** 10.1371/journal.pone.0035808

**Published:** 2012-05-21

**Authors:** Andrew L. Rhyne, Michael F. Tlusty, Pamela J. Schofield, Les Kaufman, James A. Morris, Andrew W. Bruckner

**Affiliations:** 1 New England Aquarium, Research Department, Boston, Massachusetts, United States of America; 2 Roger Williams University, Department of Biology and Marine Biology, Bristol, Rhode Island, United States of America; 3 United States Geological Survey, Southeast Ecological Science Center, Gainesville, Florida, United States of America; 4 Boston University Marine Program, Department of Biology, Boston University, Boston, Massachusetts, United States of America; 5 Conservation International, Arlington, Virginia, United States of America; 6 National Oceanic and Atmospheric Administration, National Ocean Service, National Centers for Coastal Ocean Science, Beaufort, North Carolina, United States of America; 7 Khaled bin Sultan Living Oceans Foundation, Landover, Maryland, United States of America; University of Western Australia, Australia

## Abstract

The aquarium trade and other wildlife consumers are at a crossroads forced by threats from global climate change and other anthropogenic stressors that have weakened coastal ecosystems. While the wildlife trade may put additional stress on coral reefs, it brings income into impoverished parts of the world and may stimulate interest in marine conservation. To better understand the influence of the trade, we must first be able to quantify coral reef fauna moving through it. Herein, we discuss the lack of a data system for monitoring the wildlife aquarium trade and analyze problems that arise when trying to monitor the trade using a system not specifically designed for this purpose. To do this, we examined an entire year of import records of marine tropical fish entering the United States in detail, and discuss the relationship between trade volume, biodiversity and introduction of non-native marine fishes. Our analyses showed that biodiversity levels are higher than previous estimates. Additionally, more than half of government importation forms have numerical or other reporting discrepancies resulting in the overestimation of trade volumes by 27%. While some commonly imported species have been introduced into the coastal waters of the USA (as expected), we also found that some uncommon species in the trade have also been introduced. This is the first study of aquarium trade imports to compare commercial invoices to government forms and provides a means to, routinely and in real time, examine the biodiversity of the trade in coral reef wildlife species.

## Introduction

Every year, millions of marine organisms are removed from the world’s coral reefs and associated habitats and inserted into a pipeline that empties into more than two million homes and public aquariums worldwide [1], [2]. The majority end up in the United States (U.S.), followed by Europe, Japan, and a handful of other countries. Once a cottage industry, export of marine ornamentals has grown into a major global enterprise, and can be fuelled by high profile exposure through movies such as Pixar’s ‘Finding Nemo’ [3]. Extraction occurs primarily from biodiverse coral reefs within the Coral Triangle Region, including the waters off the pacific countries of Indonesia, Malaysia, Papua New Guinea, the Philippines, Solomon Islands and Timor-Leste [4]. The most recent estimates suggest that the trade targets over 150 species of stony corals, hundreds of species of non-coral invertebrates, and at least 1,472 reef fish species from 50 families [2], [5], [6]. Since 1990, the aquarium trade has seen a shift in consumer preference from fish-only aquariums to miniature reef ecosystems [2], [7]. Because of this, collectors now draw upon the full suite of coral reef biodiversity to supply aesthetic and life-support aquarium services [7].

Collectors for the aquarium trade function as a peculiar and unprecedented type of generalist predator that targets both abundant and rare species, with a premium on both biodiversity and scarcity *per se*. Species with critical ecological roles are particularly vulnerable [7]. Exploitation of species providing critical ecosystem functions and services include herbivores that prevent the proliferation of fleshy macroalgae (e.g. urchins and topsnails) that would otherwise overgrow and kill important corals. Other target species feed on nuisance organisms such as bioeroding and encrusting sponges, colonial anemones (e.g. peppermint shrimp), and coral-eating invertebrates. Harvesting these species from the wild to supply them for the trade can lead a loss of biodiversity [2], [7], [8], overfishing associated with removal of immature fishes [2], the threat of introductions of non-indigenous species and/or diseases [9], the use of cyanide and environmentally destructive fishing practices [10], and ineffective management schemes [7], [11], [12]. This suite of challenges then calls to question the overall sustainability of the global aquarium trade. Coral reefs in many areas are under stress from poor watershed management, habitat destruction, global climate change, and other forms of overexploitation [13]. However, specimen collection for the aquarium trade could increase the value of source habitats to local economies and thus incentivize conservation [14]. This value-added benefit could also elevate awareness, appreciation, and education of the existence and plight of coral reefs and the inhabitants internationally [8], [15], thus improving sustainability in the aquarium trade at both the source and consumer ends. Such an outcome could yield an immensely greater conservation value than would the elimination of the trade. If this is the case, then can the trade provide a sustainable income source for small island economies? Can exporting and importing nations manage the trade to ensure that it is not destroying the most biodiverse area on the planet? Answers to these questions require data and currently there is only very limited information on which to base decisions. A few studies have attempted to quantify the movement of aquarium species from source to market, but these studies are built upon incomplete datasets.

### Limitations of Existing Monitoring Systems

Although multiple sources of trade data exist, not all data systems were intended for monitoring the wildlife trade. Compulsory data are maintained under federal mandates for species listed by the Convention on the International Trade in Endangered Species (CITES). Previous studies have demonstrated that CITES records can be inaccurate, incomplete, or insufficient [16], [17]. Listed species comprise a small proportion of the total ornamental aquatic animal trade, (namely stony corals, giant clams, and seahorses), and only a few studies [1], [9] have attempted to quantify the movement of non-CITES-listed aquarium species from source to market. For the U.S., the United States Fish and Wildlife Services (USFWS) is charged with inspection of wildlife shipments and maintains species-specific data of such shipments per CITES requirements in the Law Enforcement Management Information Systems (LEMIS). Non-CITES-listed fish and invertebrate species are only listed with general codes, so the data do not contain information on volume, diversity of species, or trade pathways [9]. The lack of specific data systems for recording all species exported and imported for the wildlife trade raises two questions: First, how can importing and exporting governments monitor the industry effectively? Second, how should sustainability be encouraged given the paucity of data? As coastal managers scrutinize practices of the live animal trade, including efforts to reduce risks from introduction and diseases, the need for accurate accounts of trade data increases while the current monitoring methods are static [17].

To date, information provided on shipment declarations has not been compared with associated invoices. Consequently, the utility of using LEMIS data for general wildlife trade analysis is untested. Here, for the first time, we (a) examine import records containing live **MA**rine **T**ropical **F**ish (listed under the general code MATF in the LEMIS database) for the one-year period 2004–2005, (b) provide the first accounting of the volume, biodiversity, and trade pathways for those fish species beyond the information given in volunteering reporting systems [2] and LEMIS, and (c) demonstrate that LEMIS, a system designed for import/export compliance and internal management of USFWS, is not well-suited to monitor the tropical ornamental fish trade and its impact on coral reef ecosystems.

## Materials and Methods

In the U.S., animal products require declaration prior to importation. Shipment declarations along with commercial invoices for live animal imports are submitted to and compiled by USFWS. For shipments containing a large number of non-CITES-listed species, USFWS allows consignees to list general codes on shipment declarations and append complete species-level data in the form of commercial invoices or packing slips [18]. The shipment declarations are maintained in the LEMIS database, but information specificity on declarations is limited to one category (marine ornamental fishes, MATF) for non-CITES-listed species of marine teleosts, making it impossible to determine the actual species or numbers of individual fishes in trade. The LEMIS database was not intended for use as a means of monitoring the wildlife trade. It serves to: (1) house species level data for all CITES-listed organisms as required by convention, and (2) determines manpower needed by USFWS (staffing and budgeting) at designated ports of entry.

To evaluate the diversity of aquarium species imported to the U.S., we reviewed all the shipment declarations and the attached commercial invoices (for a one year period, 2004–2005) showing the more complete species-level data. While 9,412 invoices were marked as containing MATF in the LEMIS database, we recovered 8,015 shipment declarations and their attached invoices containing MATF. Invoices were taken at face value, thus we could not control for incorrect information on these invoices. Shipment information from the declaration page along with species and quantity information from invoices were cataloged into a database. We utilized two methods of data input: manual entry or an automated optical character recognition (OCR) software (Abbyy FlexiCapture 9.0) which was customized for wildlife shipments. Input method was dependent on invoice quality and size. Manual entry was used for poor quality invoices (blurry, speckled, darkened, fonts less than six point, handwritten or extremely short invoices, less than 1/2 page), whereas all others were read using the OCR software. In both cases, species names were verified using World Register of Marine Species, FishBase, or the primary literature [19], [20]. We corrected species information when species names were misspelled, listed under a junior synonym, or listed with only the common name. The common name ‘green chromis’ was listed as three species (*Chromis atripectoralis, C. caerulea, C. viridis*) and thus was truncated to *Chromis viridis.* Fish were tagged as being ‘unknown’ when common names were used in which multiple species could be matched (e.g., unknown damsel), when exporters marked a species as ‘Assorted’ (e.g., assorted damsel), or when exporters marked species under genus only (e.g., *Chrysiptera* sp.). To remove errors from the database we compared species listed on invoices and in the database to FishBase distribution records and marked records as unknown if there were obvious mismatches. For example, a few exporters from the Indo-Pacific region listed Atlantic or Hawaiian endemic species on invoices, these were marked as uncertain. To ensure OCR errors were not made in the quantity field, we forced validation of OCR returns based on pricing information from the invoice using the following formula: Quantity * Unit Price  =  Extended Price. If price did not validate to quantity, a manual correction was then forced during data review. When total quantity on the invoice did not match the MATF value stated on the Declaration for Importation or Exportation of Fish or Wildlife (Form 3–177), all entries were double checked on the invoice to ensure mismatch error was on the declaration and not from keying errors.

**Figure 1:**
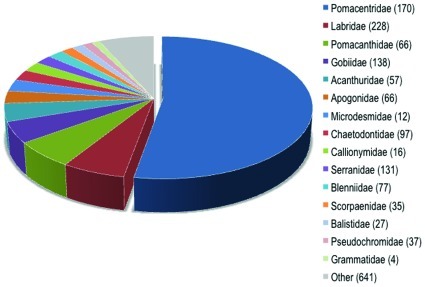
Composition by family of marine aquarium fish imported into the United States. Data for the top 20 families are provided, with the remainder grouped as ‘other’. The number proceeding the family name in the legend signifies the number of species imported within each family.

Biodiversity indices were calculated for all aggregate information, but also for each country separately. The indices calculated include Shannon’s H and Simpson’s D, along with their associated evenness scores. Both these indices characterize distributional data through a single number, the difference being that Shannon’s H is more affected by the rare species in an assemblage, whereas Simpson’s D is more subject to the dominant species in the assembly [21]. Eveness scores range from 0 (uneven, a few species make up a majority of the trade) to 1 (even, where all species occur in the trade with the same frequency). The trade data gathered through the techniques described above were then compared to biological characteristics of the species in question. To supplement the biological information, FishBase [20] was mined for information using the species identified during data extraction of the invoices. The fields collected from FishBase included trophic level, human use, minimum depth collected, maximum length, and International Union for Conservation of Nature (IUCN) Red List status. Since this paper is a description of potential trade impacts on biodiversity, the numerical FishBase data were plotted against the rank order of the volume of each species in the trade. The list of species held in public aquaria [22] were compared to those imported into the U.S. to assess the overlap between the public and home sectors of the ornamental fish trade. Finally, we sought to assess the occurrence of marine tropical ornamental fishes imported in the aquarium trade that occur as non-natives in Florida coastal waters. To do this, we overlaid reported nonindigenous species (NIS) [23] on the cumulative distribution curve of species abundance. Species were classified as ‘prevalent’ or ‘rare’ with prevalent species being those species that occur within the top 95% of total cumulative individuals imported. The number of rare species expected at random (Chi-Square test) was determined by generating random datasets (n = 1,000) of 34 species and accessing those that were classified as ‘prevalent’ or ‘rare’.

## Results

From May 2004 to May 2005 (here after 2005), marine ornamental fish entered the United States on 8,015 discrete invoices that reported a total of 11,003,181 marine fish. These more than 11 million marine aquarium fishes comprised over 1,802 species from 125 families (Fig. 1). This analysis relied on information contained on the invoices, whereas earlier estimates relied on the shipping declarations. Our detailed review of shipment invoices demonstrated that (a) the number of individual fish listed on shipment declarations matched the invoices only 52% of the time and (b) in total, volume was over-reported by 27% as shipments were often mislabeled to contain marine fish (MATF) when they in fact harbored only freshwater fish, corals, and/or other wildlife products.

Fish not identified to genus and species accounted for 4.9% of all fish (536,508 individuals), and were removed from further analyses. The total ‘known’ imports (identifiable to a species level) was 10,466,673 (Table 1). Five countries had complete identification of all individuals exported to the United States (Table 1), while fish from Ecuador and Mexico included identification of all individuals using scientific names less than 35% of the time. This is because common names were used on invoices (see USFWS 50 CFR Part 14 for regulations on declaration requirements), which makes subsequent differentiation to a genus and species level challenging. Bangladesh was excluded from Table 1, as it exported only live *Anguilla* sp. to the U.S., and these were likely not for the ornamental trade. Beyond this extraction of the invoice data, there was no way to assess the veracity of species identification on the invoices and misrepresentation could still occur, which may change the total number of species imported.

**Table 1:** The Countries that Exported Marine ornamental Fish to the U.S. in 2005.

Country	Species		Individuals				
	# sp	# indknown	% indknown	H	Eh	D	Dh
Australia	255	19,705	91.1	3.59	0.65	14.88	0.06
Bahamas	84	877	97.4	3.91	0.88	34.85	0.41
Belize	62	20,685	98	2.51	0.61	6.38	0.1
Brazil	116	29,362	81.7	3.28	0.69	14.25	0.12
Canada	44	473	70.3	3.01	0.79	10.66	0.24
Chile	3	62	100	0.89	0.81	2.08	0.69
Costa Rica	30	18,943	99.9	2.32	0.68	6.91	0.23
DominicanRepublic	52	19,534	96.4	2.04	0.52	3.17	0.06
Ecuador	29	4,686	33.9	1.24	0.37	2	0.07
Egypt	15	255	87.6	2.28	0.84	7.51	0.5
El Salvador	10	100	87	2.06	0.9	6.58	0.66
Fiji	288	165,471	88.5	3.46	0.61	13.07	0.05
FrenchPolynesia	157	46,161	67.4	2.22	0.44	3.66	0.02
Great Britain	4	10,507	100	0.65	0.47	1.48	0.37
Haiti	92	211,166	84.6	2.46	0.54	6.16	0.07
Hong Kong	6	15	93.8	1.71	0.96	5.23	0.87
Indonesia	997	3,288,434	96.2	4.41	0.64	33.55	0.03
Japan	25	195	49.5	2.49	0.77	7.2	0.29
Kenya	225	38,052	52.8	3.85	0.71	27.75	0.12
Kiribati	61	133,050	71.6	1.03	0.25	1.71	0.03
Maldives	68	12,599	93.2	3.19	0.76	17.94	0.26
MarshallIslands	101	38,319	58.5	1.89	0.41	2.82	0.03
Mauritius	62	807	42.7	3.44	0.83	18.16	0.29
Mexico	57	13,799	21.9	2.26	0.56	4.64	0.08
NetherlandsAntilles	31	2,104	100	2.55	0.74	8.66	0.28
New Caledonia	25	208	97.7	2.46	0.77	6.88	0.28
Nicaragua	43	11,273	34.9	2.68	0.71	9.9	0.23
Palau	81	13,225	55.2	2.71	0.62	7.57	0.09
Philippines	1,050	5,774,579	99	4.38	0.63	28.55	0.03
Saudi Arabia	189	62,451	92.4	3.47	0.66	18.02	0.1
Singapore	83	22,391	94.6	2.59	0.59	9.15	0.11
Solomon Islands	175	121,891	93.7	3.13	0.61	11.25	0.06
Sri Lanka	445	261,789	93.4	4.16	0.68	24.74	0.06
Taiwan	8	3,227	100	1.01	0.49	1.78	0.22
Tonga	140	10,627	84	3.65	0.74	23.86	0.17
Vanuatu	242	61,578	84.9	3.68	0.67	18.34	0.08
Venezuela	5	37	97.4	1.37	0.85	3.5	0.7
Vietnam	231	30,365	98.1	4.32	0.79	35.55	0.15
Yemen	12	17,671	100	1.11	0.45	2.02	0.17
Grand Total	1,802	10,466,673	95.1	4.81	0.64	40.78	0.02

The number and % correctly identified of both species and individuals are provided for each country, and for the aggregate total of all imports. Values are also provided for the Shannon (H) and Simpson (D) diversity indices, and their component evenness scores (E_H_ and E_D_).

Apart from the lack of precision in identifying species, another problem is that the import declaration form (a synopsis of the invoice) accompanying the invoices listed a larger number of individuals (∼15,000,000) than what was tabulated from the invoices. Furthermore, many of the declarations indicate shipments contained only marine fish (coded as MATF), when freshwater species were in fact listed on the invoices. It was common for these freshwater invoices to have source listed as wild and not captive bred, however it is unlikely that shipments of South American Cichlids exported from Singapore were wild caught fish. Likewise, clownfish exported from Great Britain were likely captive bred and not wild. It is also unknown if the *Anguilla* sp. from Bangladesh were indeed MATF, or were actually intended for the seafood trade, which seems more likely.

**Figure 2:**
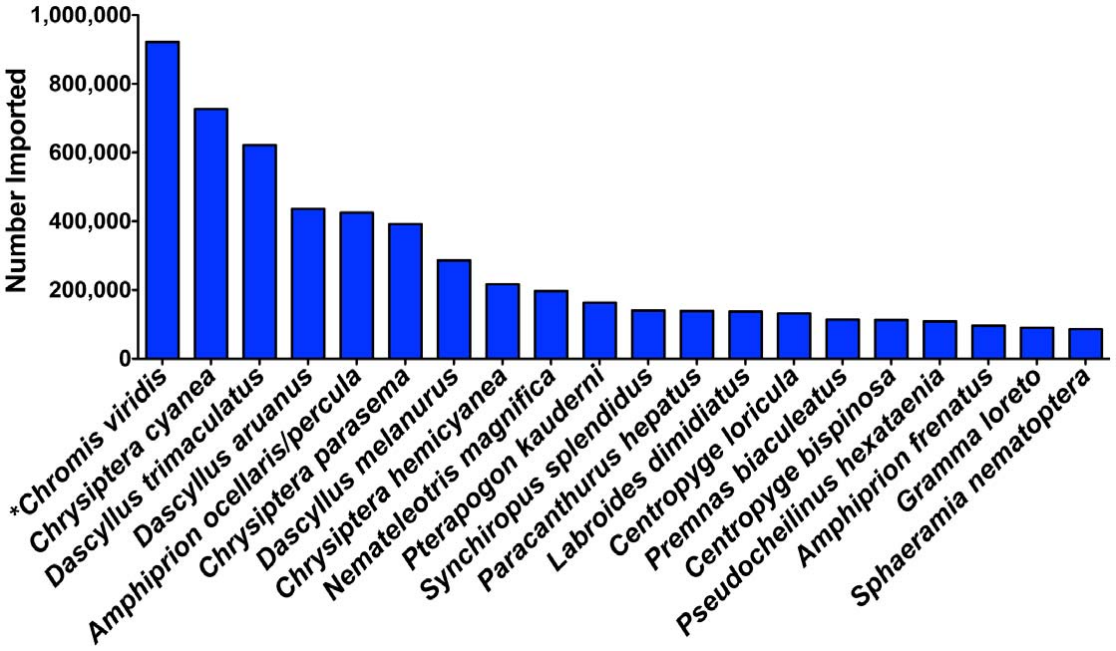
Top 20 marine aquarium fish imported into the United States. * indicate species complexes, which could represent more than one species which are all traded under the same name.

Considering the 10,466,673 individuals identified to the species level (Table 1), the vast majority of fishes imported into the U.S. were from a few teleost families (Fig. 1). Only 20 species represented 52% of the total number of individuals imported (Fig. 2). Ten of the top 20 species were damsels or anemone fishes (both Family Pomacentridae), which accounted for 76% of the individuals of this majority (Fig. 2). The top two species were *Chromis viridis* (8.8% of imports) and *Chrysiptera cyanea* (6.9% of imports; Fig. 2).

The most commonly imported species were small in body size and from shallow water habitats (Fig. S1). The first fish to occur at a minimum depth >50 m was ranked 983^rd^ (*Pseudojuloides erythrops*), whereas the first fish to exceed 2 m maximum adult total length was ranked 1050^th^ (*Cheilinus undulatus*). However, rank was not significantly associated with either minimum depth (y = 0.0025x+2.78, r^2^ = 0.02) nor maximum size (y = 0.005x+13.79, r^2^ = 0.01).

For the 1,242 species in which feeding type was indicated on FishBase, 75.5% were identified as feeding on ‘mainly animals’, while 9.5% were coded as ‘plant/detritus+animals’, and 15.5% were coded as mainly ‘plants+detritus’. As a result of the overabundance of carnivores, there was no observable pattern in trophic level of the imported fishes (y = 0.0002x+3.12, r^2^ = 0.03, Fig. S1). The highest ranked fish to exceed a trophic level of 4 was the 29^th^ ranked *Pterois volitans,* (63,284 individuals, trophic level  = 4.45).

Human Use was declared on FishBase [20] for 1,310 of the known imported species. The majority (1,023) were identified as being used in the aquarium trade, while fisheries represented 788, with gamefish (123), aquaculture (31) and baitfish (21) uses comprising the remaining categories. These were not distinct categories, and as such, 517 species were identified as being used only by the aquarium industry, 216 solely by fisheries, while 421 were used as both aquarium and fishery species. Those identified on FishBase as being used in the aquarium industry could further be identified as having commercial (989, 89.8%) or public aquarium use (112, 10.1%). However, of the 1,375 species held by public aquariums [22], 747 (54.3%) appeared on the list of species imported into the U.S. in 2005. Thus, there is a high degree of overlap between species exhibited in public aquariums, and those in the home aquarium trade.

The population status for known imported species was obtained from FishBase [20]. The majority (64.2%) were listed as not evaluated, 29.9% were of least concern, 3.1% were data deficient, and the remainder were near threatened (1.5%), vulnerable (1.1%), or endangered (0.1%). Both of the endangered species, and 14 of the 19 vulnerable species appear on the roster of species in public aquariums [22] (Table 2). A majority of the imported fish had low vulnerability, although there was no apparent association between prevalence in the trade and this metric (Fig. S1). FishBase [20] defines any vulnerability score >45 as being ‘medium to high vulnerability’. The first species to meet this definition was *Pterois volitans*, which was ranked 29^th^ most common in the trade.

**Table 2:** Marine Aquarium Species of Concern and Their Rank.

Taxon	Common Name	Rank
**Endangered**		
*Pterapogon kauderni*	Banggai cardinalfish	10*
*Cheilinus undulatus*	Humphead wrasse	1050*
**Vulnerable**		
*Cromileptes altivelis*	Humpback grouper	90*
*Balistes vetula*	Queen Triggerfish	492*
*Plectropomus laevis*	Blacksaddled coralgrouper	630*
*Lachnolaimus maximus*	Hogfish, Hog Snapper	790*
*Stegostoma fasciatum*	Zebra shark	793*
*Thalassoma virens*	Emerald wrasse	982
*Bolbometopon muricatum*	Green humphead parrotfish	1006
*Diplobatis ommata*	Oscillated electric ray	1132
*Plectropomus areolatus*	Squaretail coralgrouper	1292
*Glaucostegus typus*	Giant shovelnose ray	1312*
*Lutjanus cyanopterus*	Cubera Snapper	1385*
*Epinephelus lanceolatus*	Giant grouper	1426*
*Sanopus greenfieldorum*	Whitelined toadfish	1455
*Himantura uarnak*	Honeycomb stingray	1495*
*Nebrius ferrugineus*	Tawny nurse shark	1508*
*Rhynchobatus djiddensis*	Giant guitarfish	1521*
*Pseudanthias regalis*	High finned *Anthias*	1666
*Himantura gerrardi*	Sharpnose stingray	1768
*Rhina ancylostoma*	Bowmouth guitarfish	1793*

Rank is import volume (1 being the largest). Status derived from FishBase with an IUCN Red List Status of endangered or vulnerable. * indicates species held in public aquariums. *Hippocampus* species have been excluded from the import lists as they are CITES listed species.

**Figure 3:**
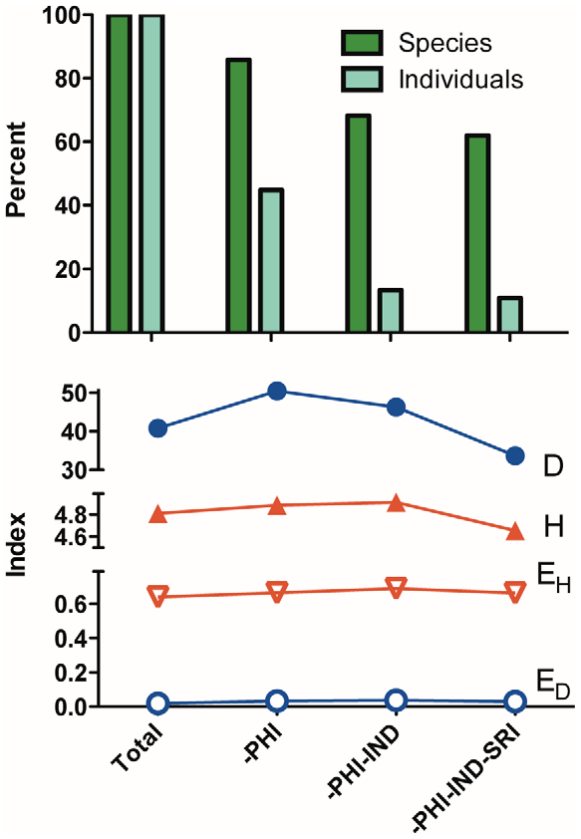
Percent Change and Diversity from Major Exporters of Marine Aquarium Fish. Top - The percent change in the number of species (black bars), and the number of individuals or marine tropical fish imported into the United States in 2005 as the most important countries, the Philippines, Indonesia, and Sri Lanka, are removed from the total. Bottom - The change in diversity (Shannon H, and Simpson D) and evenness as the same three countries are removed.

Overall, the exports of marine tropical fish to the U.S. were comprised of a relatively small number of individuals of many species. This result is supported by two observations. First, the 1,802 species indicate a high biodiversity trade, and the Shannon (H, 4.81) and Simpson (D, 40.8) diversity indices reflect this biodiversity (Table 1). The Philippines and Indonesia were the most significant export countries of marine ornamental fish to the U.S., each representing over 990 species, and 5.8 and 3.3 million fish respectively (Table 1). These two countries export twice the number of species and more than an order of magnitude more individuals than does Sri Lanka, the third largest export country. The size and importance of the top three countries is demonstrated by sequentially removing them from the total imports into the U.S. and observing a resultant decrease in both the number of species and individuals imported to 62% and 11% of the total imports (Fig. 3. top). The Philippines, Indonesia, and Sri Lanka also have the highest calculated diversity scores for any individual country. These three along with Vietnam are the only countries with Shannon diversity indices (H) >4. Indonesia, Vietnam and the Bahamas have Simpson diversity indices (D) >30.0 (Table 1).

**Figure 4:**
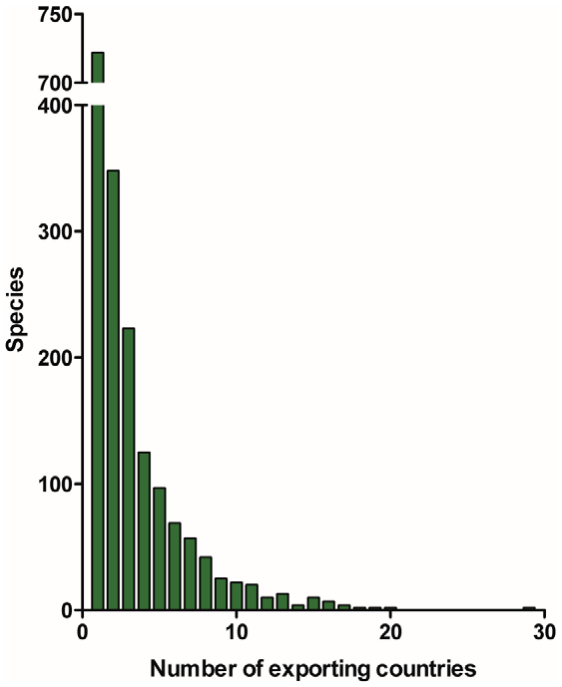
The number of countries exporting each species of marine aquarium fish into the United States. Over 700 species are exported to the United States by a single country, whereas, *Chromis viridis*, is exported by 29 different countries.

While the Philippines, Indonesia, and Sri Lanka are of great importance to the ornamental fish trade, they do not drive the diversity of the trade. If these three countries are sequentially removed from the trade data, the diversity indices of the remaining aggregated trade data drop to 4.7 and 33.7, respectively (Fig. 3. bottom). Both of these values were larger than the value for H (2.56±0.33, mean ±95% C.I.) and D (11.58±3.52) of the remaining countries averaged as separate entities. The reason that the diversity indices for the import data were greater than the component export country data is that countries tend to export a unique set of species. Nearly 750 species were exported from only a single country (Fig. 4). Conversely, *Chromis viridis* was exported from 29 countries, the most of any fish in the database (Fig. 4). In addition, only 10 or fewer individuals were imported for 326 (18.1%) of the 1,802 species that entered the U.S. in 2005.

**Figure 5:**
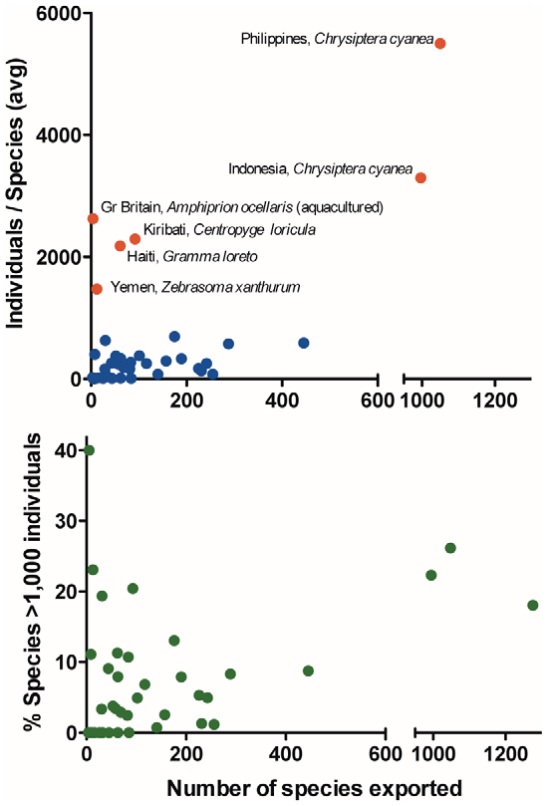
Countries and species of marine aquarium fish imported into the United States. Top: The average number of marine tropical fish individuals per species exported for the 40 countries sending fish to the U.S. in 2005. The countries exceeding an average of 1,000 individuals per species are noted, along with their most exported species. Bottom: The percent of species exceeding 1,000 individuals for each of the exporting countries.

The second way to assess that the export of aquarium fish to the U.S. comes from a small numbers of individuals representing many species is that overall, for these data, there are 5,647 unique species-country combinations of exports. Only 710 of the species-country combinations (12.6%) exceed 1,000 individuals per species, indicating that the trade consists primarily of low-volume species. Calculating the average number of individuals per species exported per country, only six countries exceed this level. These six countries include Great Britain with their most common export being *Amphiprion ocellaris* (aquaculture production, not transshipment of wild caught fish), Haiti (*Gramma loreto*), Indonesia (*Chrysiptera cyanea*), Kiribati (*Centropyge loricula*), Phillipines (*Chrysiptera cyanea*), and Yemen (*Zebrasoma xanthurum*; Fig. 5, top). Bangladesh could also be included on this list, as it exported only 1850 individuals of *Anguilla* sp. Because there are few common, but many more rare species in the trade, the evenness values for Simpson diversity index is closer to 0 than 1 (Table 1). With the exception of Great Britain, high volume species (>1,000 individuals) made up less than 25% of the number of species exported per country regardless of the total number of species exported (Fig. 5, bottom).

**Figure 6:**
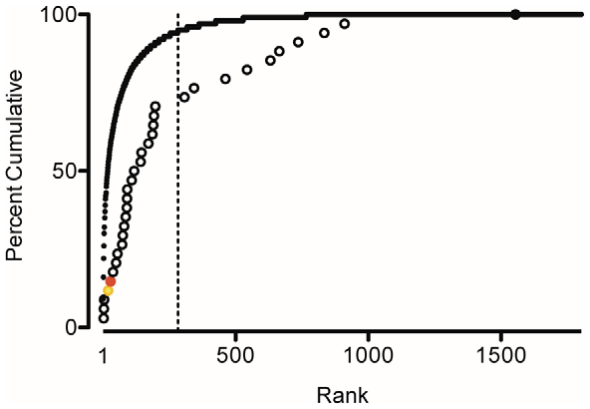
Cumulative rank of marine aquarium fish and nonindigenous species in the United States. Cumulative rank (by numbers of individuals) of marine aquarium fish species imported into the U.S. during one year 2004–2005 (solid line). Open and colored circles indicate equal weighting of occurrence for nonindigenous (NIS) species reported in marine waters of the U.S. (Florida) [23], [24]. Dotted line indicates the 95% cumulative total dividing ‘prevalent’ and ‘rare’ species. Invasive lionfish is indicated by red circle, The domestically produced Hawaiian yellow tang is indicated by a yellow circle, set at rank #5 based on Hawaiian landings (unpublished landing data).

Another concern that exists in the aquarium trade is that unwanted animals are released to the wild and thus could become invasive. Of the 1,802 species imported, 33 (1.9%) have been introduced to North America, and one (*Pterois volitans*) has become established [23], [24]. These nonindigenous species (NIS) can be divided by those that are prevalent in the aquarium trade, occur in the first 95% of the individuals in the trade, and those that are less common in the trade, remaining 5% (Fig. 6). The number of ‘rare’ species in the dataset that occur in Florida was greater than expected at random (χ^2^ = 6.47, p<0.02). Only 2 species were expected to be classified as ‘rare’, whereas 10 were observed (Fig. 6).

## Discussion

Surprisingly, the flow of biodiversity into the aquarium trade is much higher than previously estimated. The 1,802 species imported into the U.S. in 2005 was significantly greater than the previous estimate for the global trade of 1,472 species [2]. The total number of species in the global trade is likely higher than 1,802, as our data do not include those species collected inside the U.S that do not appear on import invoices (e.g. Hawaiian endemics) and those species exported but not imported into the U.S. It is unknown if those 18.1% of species in which 10 or fewer individuals were imported will occur with the same frequency each year. The diversity in the trade will likely continue to be high and will no doubt increase as new species of reef fishes are introduced into the hobby, technology improves, and aquarists share knowledge and increase husbandry skills. The marine aquarium hobby has transformed into primarily a reef aquarium hobby over the past 20 years [7], and many of the smaller fish species such as gobies, wrasses, and anthiases (small, colorful Serranidae) are becoming more popular. Additionally, there is increasing demand for uncommon organisms [25] with ‘rare’ fish commanding prices of up to U.S. $20,000 [26]. Notwithstanding the emphasis on new and rare species; the vast majority of fishes imported into the U.S. are from a few teleost families, where 20 species represent 52% of the total number of individuals imported. Of this majority, about 77.6% were damsel and anemone fishes representing 10 of the top 20 species.

Most of the species currently traded are abundant and occur over wide geographic areas and are generally not endemic or ‘rare’. One exception to this is *Pterapogon kauderni* (Banggai cardinalfish), occurring naturally only in Sulawesi. Once *P. kauderni* entered the marine aquarium trade it quickly became heavily traded and overexploited [27], [28]. Import prices of *P. kauderni* dropped rapidly as supplies increased, the species became a commodity item, and local population suffered a reduction of population fitness due to difficulty of finding mates at such low densities [29]. *P. kauderni* was subsequently transported to new localities by collectors, where it is now invasive [30] and has been implicated in the translocation of disease [31].

Another consideration bearing on the impact of the aquarium trade is the recent realization that endemism to island groups or achipelagoes has been under-estimated [32]. Population recovery can be dependent upon larvae coming from within the specific island group or from far distances. Local populations may be much more vulnerable to extirpation than previously estimated [32].

Our results indicate that prior assessments of the volume of aquarium fish traded [1], [2] are likely overestimated. Values placed on the declaration page of the import documentation matched those of the invoice in only 52% of cases examined. Additionally, volume of MATF trade was over-reported by 27%, as numerous shipments that were mislabeled as marine fish only contained freshwater fish, corals or other wildlife products. Prior estimates may underestimate biodiversity because information on the shipping declaration frequently does not match the corresponding invoice. Shipments of non-CITES-listed species are not subject to the stringent confiscation standards for paperwork violations. Importers may misreport the total number of fish and invertebrates on shipment declarations because of the uncertainty of cargo space or stock at the time when shipment declarations are prepared. This discrepancy is only evident when shipment invoices are inspected, as shipment invoices are appended to declarations at the time of importation. Furthermore, importers use invoices to prepare stock lists and holding tanks for arriving shipments. On occasion we noted comments by importers and/or USFWS inspectors (check marks by species names) on invoice copies indicating they reviewed these invoices for accuracy of species identification. It is for these reasons that the invoice is believed to be more accurate than the declaration forms. Because freshwater species appear on invoices which are encoded as ‘MATF’, previous studies [33] utilizing LEMIS have led to erroneous reports of countries exporting large numbers of marine fish to the U.S. (e.g. Singapore & Thailand).

Approximately 40 countries supply fish to the marine aquarium trade in the U.S. The data derived from this study reaffirms the prior finding [5] that the Philippines and Indonesia account for 86.6% of the imports (5,774,579 (55%) and 3,288,434 (31%) individuals, respectively). While previous data on the geographic sourcing of aquarium specimens appear more sound than those for species identification or number of specimens, there remain significant deficiencies. For example, Caribbean fisheries were not generally reported in the Global Marine Aquarium Database GMAD [2]; yet our data indicate that Haiti is one of the leading exporters of marine aquarium species to the U.S. (in 2004–2005 it ranked fourth out of 40 exporting countries).

Public aquariums significantly overlap with the home hobbyist aquarium trade, as 54% of the species held in public aquariums were imported into the U.S. in 2005. While some public aquariums collect their own fish, many source fish from commercial retail sources. The 47% of species on public display that are not on the import list are either infrequently imported, native species, inhabit cold waters (typically not kept in home aquaria), or species that are too large for home aquarists.

### Invasive Concerns

An emerging threat to marine ecosystems is the introduction of fishes from foreign locales [24], [34], [35]. For example, in Florida alone, over 30 nonindigenous species (NIS) of marine fishes have been documented in coastal waters [23]. Most of these species are from the Indo-Pacific region and are present in the aquarium trade. The pathways that could lead from the aquarium industry to foreign coastal waters include - but are not limited to - intentional dumping and natural disasters that liberate captive species. Regardless of the pathway into non-native environments, documentation of the species’ prevalence in the aquarium trade provides valuable insight. For example, many of the NIS in Florida’s coastal waters are common in the trade and are likely to exhibit high propagule pressure [36], [37], which has been demonstrated to correlate with NIS status for aquarium species [38], [39], [40]. Other NIS present in Florida which are less common in the trade, are likely to possess other life history characteristics that facilitate NIS status [41]. However, the model generating the data for life history characteristics in FishBase [42] needs to be verified, as *Pterois volitans* is rated as a high vulnerability fish, yet is a significant invasive species [24]. Thus, with the knowledge of which species or groups of species are in the trade, natural resource managers can make educated appraisals of the suite of fishes thought most likely to be invasive. As our data demonstrate, some species are making their way into nonnative habitats for reasons other than propagule pressure alone.

### Data Solutions

Recent intergovernmental panels [43] and scientists [9], [33] have called for better information sharing and reform in the wildlife trade [44]. By going beyond shipment declarations we have been able, for the first time to, provide data that can compare the popularity of species in the aquarium trade with documented reports of introductions, examine trade volumes of specific species from export to import country, and demonstrate that the utility of LEMIS and the declaration document data is limited for use in analyses of the aquarium trade. Collecting invoice data currently appears the most accurate within the bounds of trade practices. LEMIS was designed primarily to track CITES-listed species, and thus use of data for other purposes should be treated cautiously. The use of the monotypic codes (i.e. MATF) is limited in scope to track trade of non-CITES-listed species. Volunteer databases (i.e., GMAD) also provide little insight on the trade because of their limited scope spatially and temporally [45]. In moving forward, wildlife monitoring efforts may benefit by utilizing a real-time capture system of invoices rather than shipment declarations. To do so would include correcting deficiencies in the current invoice reporting framework (illegibility of documents and the inaccurate recording of genus and species data, including only listing to the genus level, the use of common names associated with multiple scientific names, and the mislabeling of fish). Furthermore, efforts could be expended to assure that invoice documentation does indeed accurately reflect shipment contents.

As the species composition and numbers of individuals imported changes with time as well as hobbyists trends, it is vital to maintain a system that captures the dynamic nature of the trade. The Optical Character Recognition (OCR) based system described herein is an effective method for capturing trade data from such an industry. The main limitations to the system’s capability for data capture are image quality (faxed copies with low resolution) and lack of standardized invoices. Requiring readable documents through electronic data submission prior to shipment importation would allow for subsequent automated capture of wildlife shipment data which could then be used to monitor the trade in real time. Only then will it be possible for industry, governments, intergovernmental agencies, and researchers to monitor and conduct knowledge-based reforms in the marine aquarium wildlife trade.

Ultimately, this system could be used to monitor the wildlife trade in real time. The software package we have developed could provide timely and accurate data that could be used to reduce the risks inherent to the wildlife trade and to protect listed species (CITES). The system could also assist wildlife inspectors with identification of thousands of species from across dozens of phyla.

### Influences of the Marine Ornamental Fish Trade on Coral Reef Fish Populations and Habitats

The 11 million coral reef fishes entering the U.S. each year is a surprisingly large number, but most coral reef fishes are highly fecund [46], [47], so we are forced to ask: is the ornamental fish trade a priority issue for coral reef conservation? While it is certainly possible that the direct take of coral reef fishes could in many instances (e.g., *P. kauderni*) pose a risk to their survival in the wild [27], [28], this does not seem a major consideration in the face of much larger stressors whose influences are not in doubt [48], [49]. There is a paucity of data regarding the stock health for the majority of fishes entering this trade (see also [44]), as the status of 64% of the species has not been assessed. While ideally all species should be appropriately managed, the priority needs to be placed on the few species that make up the bulk of the trade [7]. Coral reefs are under a host of pressures, both global (economic globalization and global climate change) and local (poor watershed management, overfishing, habitat destruction), with the result that nearly three quarters of the world’s coral reefs are already severely threatened [50]. These global pressures arise in part from global citizens’ unfamiliarity of the importance of coral reefs thereby causing an under-valuation of coral reef habitats in the wild. Greater awareness of, and appreciation for coral reefs by citizens of high-consumption nations could play a significant role in building support for reduced greenhouse gas emissions. Local anthropogenic stressors on coral reefs are driven by need – in the case of developing tropical nations, a significant need. Careful management and sustainable practices in the marine ornamental trade could help to both offset its own carbon and environmental footprint, and greatly elevate the value of sustainable coral reef habitats for the world’s inhabitants of tropical coastlines. Such a larger, nuanced view is essential if coral reefs, and the tens of thousands of species that inhabit them, are to survive the next century in a recognizable and valuable form.

## Supporting Information

Figure S1 **Size, tropic level, length and vulnerability of marine aquarium fish imported into the United States.** Data mined from FishBase for the maximum depth, the trophic level, the maximum length, and the vulnerability of the fish by rank order (volume) of imports to the United States. Tropic Level based on 2.0 detritus feeding to 4.5 feeding solely on other fish.
(EPS)
